# Bioactive Components, Untargeted Metabolomics and Bioinformatics of *Chaenomeles speciosa* Fruit on Uric Acid-Lowering Activity Assessment

**DOI:** 10.3390/foods15010020

**Published:** 2025-12-22

**Authors:** Mingzhen Zhang, Cong Liu, Yan Zhang, Zhangyaoyu Yuan, Shi Chen, Huihui Zhang, Xianju Huang, Lvyi Chen, Zhinan Mei, Yuebin Ge

**Affiliations:** 1 School of Pharmaceutical Sciences, South-Central Minzu University and Ethnopharmacology Level 3 Laboratory, National Administration of Traditional Chinese Medicine, Wuhan 430074, China; 18894224590@163.com (M.Z.); lc2023110456@163.com (C.L.);; 2 College of Plant Science and Technology, Huazhong Agricultural University, Wuhan 430070, China

**Keywords:** *Chaenomeles speciosa* (Sweet) Nakai, hyperuricemia, metabolomics, bioactive components, functional food

## Abstract

*Chaenomeles speciosa* (Sweet) Nakai (CF), a traditional food in East Asia and a recent addition to clinical dietary recommendations, has demonstrated potential for managing hyperuricemia. However, its bioactive components and therapeutic mechanisms remain largely unexplored. In this study, we used an integrative approach incorporating serum pharmacochemistry, metabolomics, bioinformatics, molecular docking, and in vitro/vivo validation to investigate CF’s effects and mechanisms in hyperuricemia. In hyperuricemic mice, CF significantly reduced serum uric acid, creatinine, and blood urea nitrogen (BUN) levels, improved kidney histopathology, and restored redox balance by increasing antioxidant enzyme activities (SOD and GSH-Px) while lowering malondialdehyde (MDA) levels. Metabolomic analysis revealed that CF modulated pathways associated with oxidative stress, including purine metabolism, arachidonic acid metabolism, and α-linolenic acid metabolism, to reverse hyperuricemia-associated metabolic perturbations. Correlation analysis between differential metabolites and serum-absorbed constituents identified androsin, cynaroside, and salicin as potential bioactive compounds. These compounds showed high predicted binding affinities to COX-1, PGE2, and XOD in molecular docking, and these interactions were validated by in vitro assays, where the compounds effectively suppressed inflammatory cytokine production and inhibited XOD activity. Overall, CF exerts anti-hyperuricemic and renoprotective effects through coordinated regulation of purine metabolism, inflammation, and oxidative stress, supporting its potential as a functional food or complementary therapy for hyperuricemia-related conditions.

## 1. Introduction

The global prevalence of hyperuricemia is on the rise, posing a significant public health challenge due to its strong bidirectional association with renal dysfunction and chronic kidney disease [1,2]. While hyperuricemia arises from an imbalance between uric acid (UA) production and excretion [3], elevated serum UA levels exacerbate kidney damage through mechanisms including inflammatory pathway activation, oxidative stress induction, and direct impairment of renal excretory function [4]. Notably, while pharmaceutical treatments can help to regulate UA levels, they are often associated with limitations such as side effects and limited efficacy. This highlights the need for alternative therapeutic approaches, such as dietary interventions, which have the potential to regulate UA levels and provide renal protection with fewer risks.

Dietary modulation represents a frontline strategy for managing serum UA concentrations: purine-rich foods augment endogenous UA synthesis and elevate serum levels, while increasing fruit intake and reducing animal product consumption may mitigate hyperuricemia risk [5,6]. Among fruits with potential therapeutic value, *Chaenomeles speciosa* (Sweet) Nakai (CF, commonly known as “ZhoupiMugua” in China)—a member of the Rosaceae family widely cultivated in East Asia [7]—has garnered attention for its nutritional and bioactive properties. Specifically, CF fruits are enriched in flavonoids, triterpenes (e.g., oleanolic acid, ursolic acid), organic acids, and tannins [8,9], which contribute to their documented anti-inflammatory, antioxidant, and renal-protective effects [10,11,12]. Critically, closely related *Chaenomeles sinensis* has demonstrated xanthine oxidase (XOD) inhibitory activity, reducing serum UA levels in hyperuricemic mice [13], suggesting the genus may harbor untapped potential for hyperuricemia management.

Reflecting this promise, CF fruit has been included in China’s Dietary Guidelines for Hyperuricemia and Gout in Adults (2024 Edition) as a recommended dietary supplement [National Health Commission of the People’s Republic of China, 2024]. However, despite its widespread consumption and preliminary evidence of efficacy, the specific bioactive compounds in CF responsible for UA-lowering effects, their molecular targets, and the metabolic pathways they modulate in vivo remain poorly defined. This knowledge gap is compounded by the multifactorial nature of hyperuricemia and the complex phytochemical profile of CF, necessitating a comprehensive, integrated investigative approach.

To address these limitations, the present study employs metabolomics, bioinformatics, and in vivo and vitro validation to dissect the therapeutic mechanisms of CF fruit in a hyperuricemia model. Our objectives are threefold: (1) identify CF-derived bioactive compounds; (2) evaluate the effects of CF on hyperuricemia and renal injury; (3) explore the molecular mechanisms and targets of CF. This integrated strategy will provide novel mechanistic insights into CF’s role as a functional food for hyperuricemia management and related renal complications.

## 2. Materials and Methods

### 2.1. Preparation of C. speciosa Fruits Extracts

*Chaenomeles speciosa* fruit (CF) was provided by Qilingtou Mugua planting base (Changyang, China) and authenticated by Prof. Xinqiao Liu from school of pharmaceutical science, South-Central Minzu University. 90 g of CF sample was decocted with water (1:10, *w*/*v*) for 40 min. The extraction was repeated twice. The collected solution was filtered and concentrated to 50 mL volume for experiments. Extraction yield (17.73 ± 1.86%, *n* = 3 batches) and final extract concentration was 1.8 g/mL.

### 2.2. Reagents

Allopurinol (ALL) was acquired from Hefei JiuLian Pharmaceutical Co., Ltd. (Hefei, China, Batch No. 20220905). Potassium oxonate (PO, purity ≥ 98%), hypoxanthine (HX, purity ≥ 99%), monosodium urate (MSU), androsin and cynaroside were obtained from Shanghai Yuanye Biochemical Co., Ltd. (Shanghai, China, Cat. No.: S17112, S18025, S30775, B20131, B20887). Salicin was acquired from Shanghai Aladdin Biochemical Technology Co., Ltd. (Shanghai, China, Cat. No.: S104923). Assay kits of xanthine oxidase (XOD), blood urea nitrogen (BUN), uric acid (UA) and creatinine (CRE) were purchased from Nanjing Jiancheng Bioengineering Institute (Nanjing, China, Cat. No.: A002-1-1, C03-1-1, C012-2-1, C011-2-1). Malondialdehyde (MDA), superoxide dismutase (SOD), and glutathione peroxidase (GSH-Px) were acquired from Beyotime Biotechnology Co., Ltd. (Shanghai, China, Cat. No.: S0131S, S0109, S0057S). Cell counting kit-8 (CCK-8) were acquired from Dojindo Laboratories (Kumamoto, Japan, Cat. No.: CK04). ELISA kits for prostaglandin E2 (PGE2) and cyclooxygenase-1 (COX-1) was purchased from Beijing Solarbio Science & Technology Co., Ltd. (Beijing, China, Cat. No.: SEKM-0173) and Shanghai Jianglai Biotechnology Co., Ltd. (Shanghai, China, Catalog No.: JL13697). ELISA kits for interleukin-1β (IL-1β) and tumor necrosis factor-α (TNF-α) were purchased from Abclonal Co., Ltd. (Wuhan, China, Cat. No.: RK00027, RK00006). Lipopolysaccharides (LPS) were acquired from Sigma-Aldrich (St. Louis, MO, USA, Cat. No.: L2630).

### 2.3. Compositional Analysis of CF

1.0 g CF watery extract was added with 40 mL 80% methanol and ultrasonicated for 30 min. Suspension was centrifuged at 12,000 rpm for 10 min under 4 °C, then 100 μL of supernatant was pipetted into an injection vial for detection. Sample separation was performed using a Vanquish Flex UHPLC system (Thermo Fisher Scientific, Inc., Waltham, MA, USA) equipped with an ACQUITY UPLC HSS T3 column (Waters Co., Milford, MA, USA). HPLC conditions: mobile phase A (water + 0.1% formic acid, *v*/*v*)/B (acetonitrile, *v*/*v*); flow rate 0.3 mL/min, column temp 40 °C, injection 6.0 μL. Gradient: 98% A (0–1.0 min) → 70% A (14.0 min) → 0% A (25.0–28.0 min) → 98% A (28.1–30.0 min), linear transitions. The MS data was collected by a hybrid quadrupole orbitrap mass spectrometer (Q Exactive, Thermo Fisher Scientific, Inc., Waltham, MA, USA) equipped with a HESI-II spray probe. The data was acquired in “Full scan/dd-MS2” mode. Full scan parameters were set as follows: resolution 7000, automatic gain control (AGC) target 1 × 10^6^, mass-to-charge ratio scanning range: 100–1500. The dd-MS2 data was collected with the parameters of resolution 17,500, auto gain control target 1 × 10^5^, maximum isolation time 50 ms, loop count of top 10 peaks, isolation window *m*/*z* 2, collision energy 10 V, 30 V, 60 V and intensity threshold 1 × 10^5^. Metabolites were identified via a multi-dimensional comprehensive evaluation by searching the reference standard database (TCM Pro 2.0, Hexin Technology Co., Ltd., Beijing, China) and an in-house theoretical compound library. The in-house library was constructed by integrating published data from Beijing University of Chinese Medicine Herbal Compound Identification Database, TCMSP, TCMID, and literature-reported constituents of the studied herbal formula. Identification was based on multiple orthogonal criteria: retention time deviation (<±0.2 min), precursor ion mass error (<5 ppm), MS/MS fragment pattern similarity (>85%), isotope distribution consistency, and relative peak intensity.

### 2.4. Animal Experiments

Specific pathogen-free (SPF) male KM mice (6–8 weeks old, 18–22 g) were purchased from Hubei Provincial Center for Disease Control and Prevention [License No. SCXK (E) 2020-0018, SYXK 2021-0089, Wuhan, China]. Before the experiment initiation, the mice were allowed one-week environmental adaptation period to recover from transportation stress and adapt to the housing conditions. Animals were housed 6 per cage (sufficient activity spaces, terile wood shavings bedding, changed twice weekly) in an SPF laboratory under a 12 h light–dark cycle (23 ± 3 °C, 40–70% humidity) with standard chow (purchased from Wanqian Jiaxing Biotechnology Co., Ltd., Wuhan, China) and water ad libitum. All mice were randomly assigned to each group using a random number table. Administration and all outcome assessments were performed double-blind to eliminate experimenter bias. The study was approved by the Committee on Research Ethics and Technology Security, South-Central Minzu University (Approval No. 2022-scuec-057, Approval date: 2 November 2022, Wuhan, China). All procedures were conducted in accordance with Chinese legislation on the use and welfare of animals.

48 mice were divided into 6 groups randomly, namely the control group (CON), the model group (PO + HX, 300 mg/kg + 500 mg/kg, allopurinol group (ALL, 20 mg/kg), CF extract groups including low (CF-L, 0.8 g/kg), medium (CF-M, 1.6 g/kg) and high (CF-H, 3.2 g/kg). With the exception of the control group (injections an equal volume of normal saline), all mice were administered intraperitoneal injections of PO and gavaged HX to establish a hyperuricemia model. Both PO and HX were suspended in 0.5% CMC-Na. The mice were orally administered with the positive and tested samples 1 h later. The control and model groups were administered an equivalent volume of saline via oral gavage. Experiments were conducted daily in the morning in an SPF-grade operating room for 14 days. During the experiment, the occurrence of toxic or lethal events in mice will be observed.

### 2.5. Blood and Tissues Sample Collection

After 14 days of treatment, the mice were weighed, anesthetized, and subsequently euthanized by CO_2_ asphyxiation to obtain blood samples via enucleation for further analysis. Whole blood was on standing for two hours, then centrifuged at 3000 rpm for 10 min under 4 °C to collect serum. Simultaneously, liver and kidney tissues were rapidly isolated and rinsed with 0.9% saline solution. The left kidney was fixed for histopathological analyses (final n = 6/group after quality control), whereas the right kidney and whole liver were snap-frozen in liquid nitrogen and stored at −80 °C until biochemical and metabolomics assays.

### 2.6. Biochemistry and Histopathology Detection

Biochemical markers including XOD, BUN, UA, CRE, MDA, SOD, GSH-Px and PGE2 were measured according to the instructions of the kit. The detection wavelengths and principles are followings: UA (510 nm, enzymatic colorimetric method), CRE (546 nm, sarcosine oxidase method), BUN (520 nm, diacetyl monoxime colorimetric method), XOD (530 nm, colorimetric method), MDA (532 nm, TBA-based method), SOD (560 nm, NBT photoreduction method), PGE2, TNF-α and IL-1β (450 nm, sandwich ELISA). All assays were run in duplicate, and standard curves showed linearity with R^2^ > 0.990. Target-wavelength OD values were measured for samples, blank-subtracted to eliminate background interference, and used for subsequent concentration and activity quantification.

Renal tissues were fixed with 4% paraformaldehyde at 4 °C overnight, embedded in paraffin, and cut into 4-μm sections. Hematoxylin-eosin staining was performed on the prepared sections. The stained sections were observed and photographed under a 200× light microscope (Olympus, Tokyo, Japan). Subsequently, semi-quantitative assessment of key pathological parameters was performed [14,15].

### 2.7. Identification of Blood Components

A total of 100 μL of serum sample was added to 300 μL pre-cooled chromatographic grade methanol. After centrifugation at 12,000 rpm for 10 min at 4 °C using a refrigerated centrifuge, 270 μL of supernatant was carefully collected without disturbing the precipitate. The supernatant was concentrated by vacuum centrifugation for 4 h, with sealed centrifuge tubes to prevent sample loss. Subsequently, 90 μL of 50% methanol aqueous solution was added, followed by vortexing at 800 rpm for 1 min and centrifuged again at 12,000 rpm for 10 min at 4 °C. HPLC and MS conditions are the same as in Section 2.3 (Supplementary Tables S1–S4).

### 2.8. Biological Information Analysis

The SwissTargetPrediction database (http://www.swisstargetprediction.ch/, accessed on 22 October 2024) and PubChem (https://pubchem.ncbi.nlm.nih.gov/, accessed on 22 October 2024) were used to retrieve the predicted targets of CF’s blood-entering constituents. After deduplicating predicted targets, core targets were identified by applying a threshold of Tanimoto similarity score (T-value) > 0.12. Visualization of protein–protein interaction (PPI) networks was performed using the STRING database (https://cn.string-db.org/, accessed on 22 October 2024) and visualized via Cytoscape software (version 3.8.0) to explore the interrelationships between the active compounds and core targets involved in hyperuricemia treatment.

KEGG and GO enrichment analyses were conducted using the ClusterProfiler package (version 4.14.4) in R. Functional terms and pathways with both *p* value < 0.05 and adjusted *p* value (padj) < 0.05 were considered significantly enriched.

### 2.9. Untargeted Metabolomics Analysis

Serum samples from the control, model, and high-dose groups were subjected to metabolomic analysis. After thawing on ice, 100 μL of serum was mixed with 300 μL of pre-cooled methanol, vortexed for 1 min, and centrifuged at 12,000 rpm for 10 min at 4 °C. A 300 μL aliquot of the supernatant was collected, lyophilized under vacuum, and reconstituted in 100 μL of 50% methanol using sonication in an ice-water bath. The mixture was centrifuged again, and 90 μL of the final supernatant was transferred to vials for analysis. Each sample was injected at a volume of 5 μL.

Chromatographic separation was performed using a Waters UPLC HSS T3 column (2.1 mm × 100 mm, 1.8 μm) maintained at 40 °C. Mass spectrometry was conducted on a UHPLC-Q-Orbitrap HRMS system (Q Exactive™, Thermo Fisher Scientific), operating in both full MS and data-dependent MS^2^ (dd-MS^2^) modes. Key settings included a resolution of 70,000 for full MS and 17,500 for MS^2^, AGC targets of 1 × 10^6^ and 1 × 10^5^, a maximum injection time of 50 ms, and stepped collision energies of 10, 30, and 60 eV. Up to 10 precursors per cycle were selected for fragmentation with dynamic exclusion. Additional LC and MS parameters are provided in Supplementary Tables S4–S7.

Quality control (QC) samples were prepared by pooling aliquots from all study groups and were injected at regular intervals to monitor instrument stability. Ion features with a coefficient of variation (CV) > 15% in QC samples were excluded from further analysis (3504 features retained). Retention time alignment and total ion current (TIC) normalization were performed using Progenesis QI software. Metabolite identification was based on accurate mass and MS^2^ spectral matching against the Human Metabolome Database (HMDB) and lipid databases such as LipidMaps.

### 2.10. Molecular Docking

Three bioactive compounds were selected for molecular docking based on serum component analysis and bioinformatics predictions. The crystal structures of COX-1 (PDB ID: 1EQG), XOD (1FIQ), and PGE2 (PDB ID: 2ZB4) were obtained from the RCSB Protein Data Bank (http://www.rcsb.org/, accessed on 2 May 2025). Co-crystallized ligands were retained for redocking validation, and water molecules were removed using PyMOL 2.6.0.

Ligand structures were retrieved from the PubChem database and converted to 3D format. Geometry optimization was performed using the MMFF94 force field in Open Babel. Proteins and ligands were prepared in AutoDock Tools (version 1.5.7), including hydrogen addition and conversion to PDBQT format. Docking simulations were performed using AutoDock Vina with an exhaustiveness value of 10. The grid box parameters are provided in the Supplementary Table S10. Known inhibitors such as indomethacin (for COX-1 and PGE2) and febuxostat (for XOD) were used as positive controls. Docking results were visualized using PyMOL.

### 2.11. BMDM & BRL3A Cell Culture

Bone marrow-derived macrophages (BMDM) were isolated from the bone marrow of the femur and tibia of C57BL/6J mice. After euthanasia, the mice were sterilized by immersing in 75% ethanol, then femurs and tibiae were stripped in a sterile environment and rinsed in pre-cooled phosphate-buffered saline (PBS). Bone marrow cells were rinsed with 2% fetal bovine serum (FBS) and released, and the cell suspension was filtered through a 70 μm filter, centrifuged to collect the cells, and lysed in erythrocyte lysate for 1 min and then centrifuged again. The cell sediment was resuspended in dulbecco’s modified eagle medium (DMEM) containing 10 ng/mL macrophage colony-stimulating factor (M-CSF), 10% fetal bovine serum, 1% penicillin-streptomycin. Buffalo Rat Liver-3A (BRL3A) cells are fibroblast-like cell lines isolated from the liver of Buffalo rats. The BRL3A cells line was purchased from Procell Life Science & Technology Co., Ltd. (Wuhan, China). The cells were grown in DMEM supplemented with 10% fetal bovine serum and 1% penicillin-streptomycin. BRL3A cells at passages 3–10 were used for all experiments. Both BMDM and BRL3A cells were cultured at 37 °C and 5% CO_2_ in a humid environment.

### 2.12. Cell Viability Assay

Cytotoxicity of the three key compounds was detected using CCK-8 assay. After BMDM was fully differentiated and BRL3A cells grew to logarithmic phase, BMDM and BRL3A cells were inoculated in 96 microtiter plates (cell concentration 6 × 10^4^/mL) for overnight. After treatment with different concentrations of androsin, cynaroside and salicin (1, 5, 10, 25, and 100 μM) for 24 h, 10 μL of CCK-8 was carefully added to each well to avoid air bubbles. The absorbance at 450 nm was measured after 1–2 h of incubation using an enzyme labeller (Multiskan SkyHigh, Thermo Scientific, USA).

### 2.13. Establishment of Cell Models and Indicator Detection

BMDM cells were inoculated in 96-well plates at 6 × 10^4^/mL, pre-treated by adding different concentrations of androsin, cynaroside and salicin for 24 h, with MCC950 as a positive control. After stimulation with LPS (400 ng/mL) for 3 h, the supernatant was discarded, and medium containing MSU (400 μg/mL) was added to stimulate the cells for 6 h. Cell supernatants were collected, and the levels of IL-1β and TNF-α in cell supernatants were determined using assay kit.

BRL3A cells were inoculated into 24-well plates at a concentration of 3 × 10^5^ /mL. After 16 h of inoculation, the cells were treated with 1 mM xanthine and different concentrations of androsin, cynaroside and salicin, with Allopurinol as a positive control. The cell protein was extracted 48 h later, and the activity of XOD enzyme was detected by using XOD kit.

### 2.14. Statistical Analysis

Data were analyzed using GraphPad Prism 9.0 and SPSS 26.0 software. Quantitative data are presented as mean ± standard deviation. Prior to statistical analysis, the Shapiro–Wilk test was used to assess the normality of data distribution, and Levene’s test was used to evaluate the homogeneity of variances. For comparisons between two groups, a two-tailed independent-samples *t*-test was applied when the data met the assumptions of normality and equal variances; otherwise, the Mann–Whitney U test was used. A *p*-value < 0.05 was considered statistically significant. Graphs were generated using GraphPad Prism 9.0, with error bars representing mean ± standard deviation. Sample sizes (n) for each group are indicated in the figure legends and in the Results section.

## 3. Results

### 3.1. Identification of CF Components

**Figure 1:**
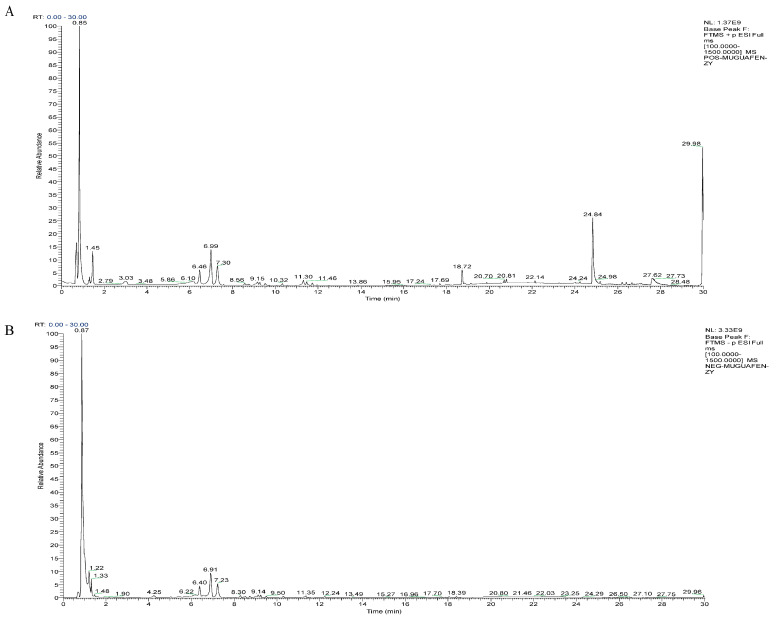
The base peak ion chromatogram of CF detected in positive and negative mode. (**A**) ESI (+). (**B**) ESI (−).

The base peak ion flow chromatograms of CF detected in positive and negative modes are shown in Figure 1. Chemical compounds such as flavonoids, terpenoids, phenolics, organic acids, glycosides, phenylpropanoids, amino acids, alkaloids, and other classes were analyzed for different content levels in CF. Flavonoids, terpenoids, phenolics, and organic acids exhibited a distinctly high proportion among them. Terpenes mainly included oleanic acid, deacetylasperulosidic acid methyl ester, linalool, asiatic acid, and bayogen. Flavonoids, for example, catechin, L-epicatechin, althosanin, rutin, hyperoside, astipine, hyperoside aglycone, kaempferol, and quercetin were found. Organic acids comprising quinic acid, citric acid, shikimic acid, isocinnamic acid, and p-coumaric acid. Phenols included Salicin, 3,4-Dihydroxybenzoic acid, Protocatechualdehyde, Isovanilline, Sinapyl alcohol, etc. Table 1 contains detailed information, with compounds sorted by relative peak area. The top 10 components with the highest abundance accounted for 89.3% of the total peak area.

### 3.2. Effect of CF on Serum UA, CRE, and BUN Levels

**Figure 2:**
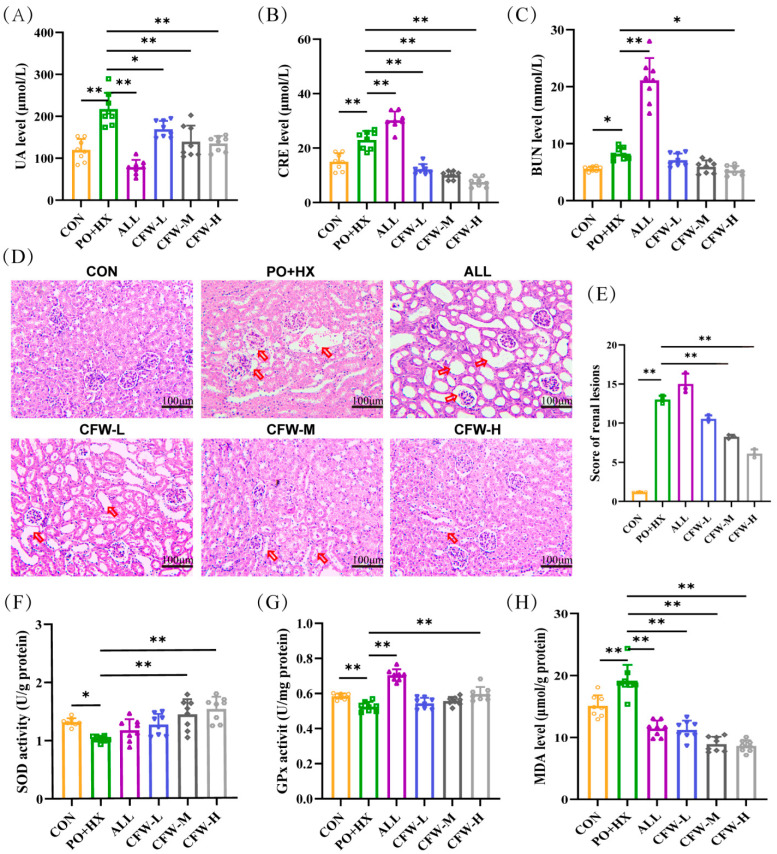
Results of biochemical indices of CF in HUA mice. (**A**–**C**) Serum levels of uric acid (UA), creatinine (CRE), and blood urea nitrogen (BUN) in different groups (n = 8). (**D**–**E**) Representative photomicrographs of kidney sections stained with hematoxylin and eosin (scale bar: 100 μm; magnification: ×200) and quantitative analysis of renal lesion scores based on H&E staining. The red arrows indicate pathological injuries, including renal tubular dilation and glomerular atrophy. (**F**–**H**) Activities of SOD (**F**) and GPx (**G**), and MDA content (**H**) in the liver (n = 8). Data are expressed as mean ± SD. * *p* < 0.05 and ** *p* < 0.01 between the indicated groups.

Many studies have confirmed that the PO + HX combination model is better than the single inducer [16,17] and no deaths or overt signs of acute toxicity (e.g., lethargy, piloerection, or reduced food intake) were observed in the experiment. The data for UA, CRE, and BUN are shown in Figure 2A–C, CF dose-dependently and significantly ameliorated hyperuricemia and renal dysfunction in the model. The PO + HX model group displayed serum UA, creatinine, and BUN levels of 216.75 ± 82.97 μmol/L, 21.78 ± 3.86 μmol/L, and 7.96 ± 1.26 mmol/L, respectively. Oral administration of CF produced dose-dependent reductions: CF-L, CF-M, and CF-H decreased UA by 25%, 29.6%, and 32.9% (to 162.78 ± 37.45, 152.65 ± 60.11, and 145.02 ± 32.13 μmol/L; *p* < 0.01); CRE by 45%, 55%, and 59% (to 12.30 ± 2.59, 10.40 ± 5.35, and 9.73 ± 2.61 μmol/L; *p* < 0.01); and BUN by 6% (ns), 19% (ns), and 34% (to 7.61 ± 2.44, 6.62 ± 2.60, and 5.54 ± 1.39 mmol/L; *p* < 0.05 only for CF-H). The ALL group showed higher CRE/BUN than the model, which aligns with clinical reports of ALL’s potential nephrotoxicity [18,19,20]. These marked reductions in serum UA, CRE, and BUN are biologically and clinically significant, as they restore UA below the crystallization threshold, normalize glomerular filtration, and effectively reverse hyperuricemia-induced renal tubular injury and inflammation.

### 3.3. Effect of CF on Renal Histopathological Change

As shown in Figure 2D,E and Supplementary Table S10, the CON group exhibited normal renal structure with clear glomerular boundaries and no inflammatory cell infiltration (total injury score 1.175 ± 0.035). In the model group, HE staining revealed renal edema, marked dilation of the renal tubular lumen, glomerular atrophy with basement membrane thickening, and obvious inflammatory cell infiltration (total injury score 13 ± 0.707, *p* < 0.001 vs. CON).

ALL group further increased the histopathological injury score to 15.5 ± 1.414 (*p* < 0.05 vs. model), this situation was consistent with the literature reported aggravated renal pathology description [19,20]. In contrast, CF treatment dose-dependently reduced pathological alterations: the low, medium, and high-dose CF groups showed total injury scores of 10.55 ± 0.636, 8.4 ± 0.141, and 6.15 ± 0.778, respectively (CF-M and CF-H *p* < 0.01 vs. model), with the high-dose group approaching the normal histological appearance of the CON group. After CF treatment, the pathological alterations in model mice improved.

### 3.4. Effect of CF on Antioxidant Activity

Liver samples were examined to assess the antioxidant properties of CF by measuring the activities of SOD and GSH-Px, along with the levels of MDA. Figure 2F shows that the SOD concentration in the model group is significantly under that in the CON group (1.32 ± 0.07 vs. 1.02 ± 0.05 U/g, *p* < 0.05). Conversely, SOD levels were elevated in the CF-M and CF-H groups (1.44 ± 0.26 and 1.53 ± 0.20 U/g, *p* < 0.01). In Figure 2G, compared with the model group (0.52 ± 0.03 U/mg), the CF-H group (0.59 ± 0.04 U/mg, *p* < 0.01) showed a significant increase in GSH-Px. The MDA concentration in the model group (19.17 ± 2.53 μmol/g) was markedly (*p* < 0.01) higher CON group (15.08 ± 1.73 μmol/g) in Figure 2H. However, the CF group significantly regulated MDA levels (11.21 ± 1.5, 8.9 ± 1.18, and 8.6 ± 0.94 μmol/g, *p* < 0.01). The elevated MDA levels, together with the decreased activities of SOD and GSH-Px, collectively indicated the occurrence of oxidative imbalance in hyperuricemic mice. CF treatment significantly restored antioxidant enzyme activities and decreased MDA levels. Importantly, these biochemical improvements in antioxidant capacity were consistent with the renal histopathological findings.

### 3.5. Joint Analysis of Absorbed Components and Bioinformatics

**Figure 3:**
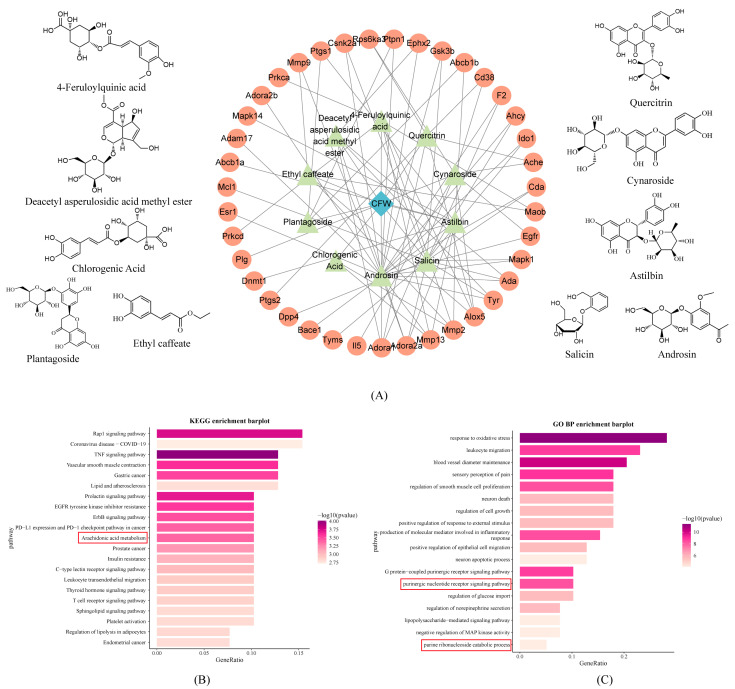
Biological information analysis of constituents absorbed into the blood. (**A**) Structural formula and PPI bioinformatic analysis of CF blood entry components, blue diamond indicates chaenomeles speciosa fruits, green triangles indicate entry components, and orange circles indicate potential targets for each entry component. (**B**) KEGG analysis of potential target of CF. (**C**) GO BP analysis of potential target of CF The red box contains the KEGG pathways and GO biological processes associated with arachidonic acid and purine metabolism.

LC-MS analysis of the absorbed constituents identified 12 prototype components, such as androsin, chlorogenic acid, cymaroside, quercitrin, and salicin, along with 12 secondary metabolites (Supplementary Figure S1, Table S8). Notably, these prototype components were consistent with the composition of the CF aqueous extract. Based on their presence in the blood and aqueous extract, these prototype compounds were selected for network pharmacology analysis. They were screened using the SwissTargetPrediction database (probability > 0.12), yielding 10 key components and 39 non-redundant predicted targets. Subsequently, a protein–protein interaction (PPI) network was constructed using the STRING database and visualized in Cytoscape software version 3.8.0, alongside the chemical structures of key components (Figure 3A).

Pathway enrichment analysis was performed using the ClusterProfiler package in R. Significantly enriched items were defined as those with both *p* value < 0.05 and adjusted *p* value (padj) < 0.05 after Benjamini–Hochberg correction, resulting in 159 KEGG pathways and 984 GO biological processes (Figure 3B,C). KEGG analysis revealed that CF may exert anti-inflammatory effects by modulating pathways such as arachidonic acid metabolism. GO analysis highlighted CF’s involvement in oxidative stress regulation and purine nucleotide receptor-mediated signaling pathways. These findings provide insights into the molecular targets and pathways regulated by CF in hyperuricemia.

### 3.6. Regulatory Effect of CF on Serum Metabolic Homeostasis in Hyperuricemic Mice

**Figure 4:**
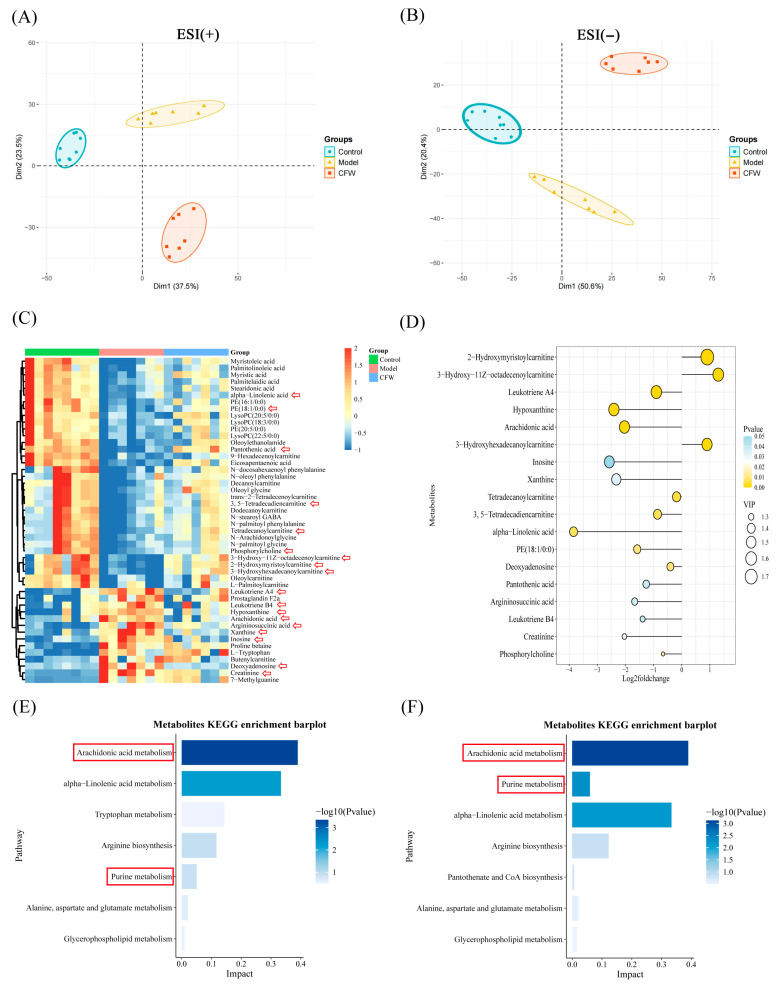
Metabolomics data analysis. (**A**) Positive ion PCA score plot for each group (n = 7). (**B**) Negative ion PCA score plot for each group (n = 7). (**C**) Heatmap of differential metabolites between groups (n = 7). Red arrows mark the metabolites displayed in the lollipop plot. (**D**) The lollipop plot illustrates the *p* values, Log_2_ fold change values, and VIP scores of these metabolites (ranked by VIP value), based on the comparison between the model group and the CF-treated group. In this analysis, a Log_2_ fold change < 0 indicates that the metabolite level was downregulated after CF treatment, whereas a Log_2_ fold change > 0 reflects an upregulation in response to CF treatment. (**E**) Analysis of metabolic pathways in the control and model groups containing differential metabolites. (**F**) Pathway analysis of different metabolites in the model and CF groups The red arrows denote metabolites exhibiting significant differences. The red box indicates the KEGG pathways related to arachidonic acid and purine metabolism.

Serum untargeted metabolomics analysis was conducted using UHPLC-Q-Orbitrap HRMS to identify metabolic alterations associated with hyperuricemia and evaluate the metabolic modulation induced by CF treatment (Supplementary Figure S2). Metabolites were identified using Progenesis QI software to process the collected data. Principal component analysis (PCA) revealed distinct separation among the control, model, and CF-treated groups in both positive and negative ion modes (Figure 4A,B), with the CF group clustering closer to the control group, indicating a partial metabolic rebalancing. To further characterize the metabolic changes, OPLS-DA was employed, yielding robust models with good explanatory and predictive power (positive ion mode: R^2^Y(cum) = 0.983, Q^2^(cum) = 0.784; negative ion mode: R^2^Y(cum) = 0.985, Q^2^(cum) = 0.837), validated through 200-time permutation tests (Supplementary Figure S3).

A total of 48 differential metabolites were identified using the criteria of VIP > 1 from the OPLS-DA model and *p* < 0.05 from Student’s *t*-test (Supplementary Table S9, Figure 4C). Among them, key metabolites dysregulated in the model group—including arachidonic acid, leukotriene A4, hypoxanthine, xanthine, and alpha-linolenic acid—were significantly reversed by CF treatment (Figure 4C,D). These metabolites are closely associated with inflammation, oxidative stress, and purine metabolism. KEGG pathway enrichment analysis highlighted arachidonic acid metabolism, purine metabolism, and alpha-linolenic acid metabolism as the top pathways impacted by CF intervention (Figure 4E,F). Notably, several key metabolites involved in inflammatory lipid signaling and purine metabolism were significantly altered in the model group and partially restored following CF treatment. In particular, arachidonic acid and leukotriene A4—pro-inflammatory eicosanoids derived from the arachidonic acid cascade—were markedly elevated in hyperuricemic mice but were significantly reduced after CF administration (Figure 4C,D), suggesting an attenuation of eicosanoid-mediated inflammatory responses. Similarly, the levels of hypoxanthine and xanthine, central intermediates in purine degradation that contribute to ROS production through xanthine oxidase, were decreased after treatment, consistent with the observed decreased uric acid. Moreover, CF increased metabolites such as phosphocholine and creatinine, which may indicate improvements in membrane phospholipid turnover and cellular energy metabolism. These metabolic adjustments align with the restored activities of antioxidant enzymes (SOD and GSH-Px) and reduced MDA levels, collectively supporting that CF confers renoprotective effects by mitigating oxidative stress and modulating key metabolic pathways, including arachidonic acid metabolism and purine catabolism.

### 3.7. Integrated Analysis of Metabolic Networks and Blood-Absorbed Constituents-Metabolite Correlations

**Figure 5:**
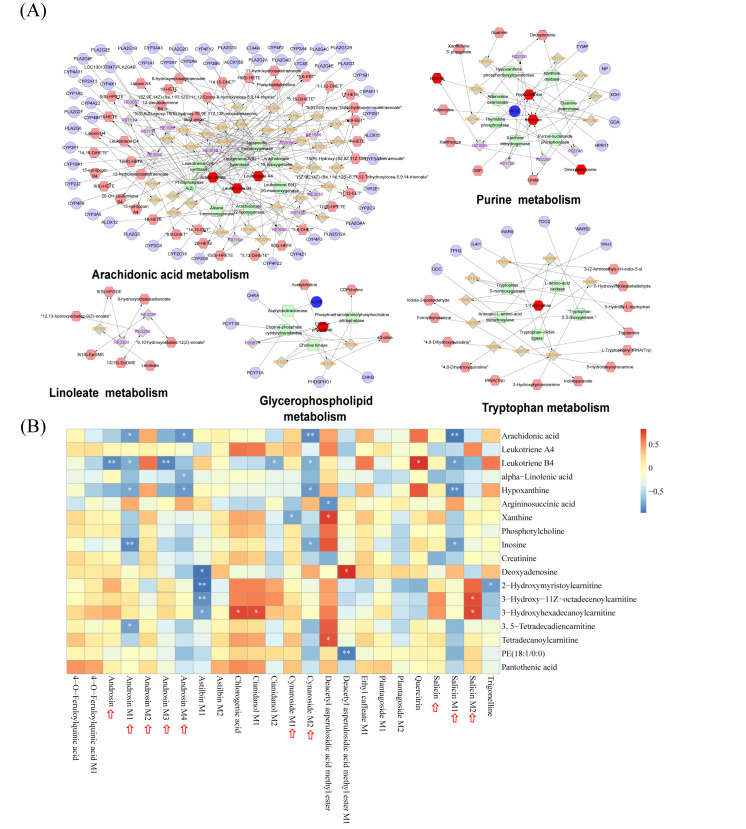
Integrated analysis of metabolic pathways and component-metabolite correlations. (**A**) Interaction networks of key metabolic pathways, including arachidonic acid, purine, linoleate, glycerophospholipid, and tryptophan metabolism. In the network diagrams, circles, diamonds, hexagons, and rounded rectangles represent genes, reactions, metabolites, and proteins, respectively. (**B**) Heatmap visualizing the correlation analysis between the constituents of CF absorbed into the blood (X-axis) and differential endogenous metabolites (Y-axis). The color scale indicates the correlation coefficient (red for positive correlation, blue for negative correlation). * *p* < 0.05 and ** *p* < 0.01 indicate statistical significance The red arrows indicate blood components and their secondary metabolites exhibiting significant correlations.

To systematically visualize the metabolic interactions modulated by CF, we constructed an integrated networ based on the identified differential metabolites and the predicted targets of the blood-absorbed constituents. The network topology unveiled the complex interplay among upstream genes, enzymatic reactions, and downstream metabolites. As shown in Figure 5A, the identified CF-regulated metabolite, specifically arachidonic acid, leukotriene A4, hypoxanthine, and xanthine, occupied central positions within the arachidonic acid and purine metabolism pathways. Serving as key metabolic nodes, their prominence highlights these pathways as critical targets potentially regulated by CF.

To further elucidate the potential “material basis” responsible for these metabolic regulations, we performed a Pearson correlation analysis between the 24 absorbed constituents (including prototype components and secondary metabolites) of CF and the 18 key differential metabolites identified by serum untargeted metabolomics (Figure 5B). The heatmap revealed significant correlations between specific prototype components and the restoration of metabolic markers. Notably, salicin, cynaroside, androsin and its metabolites exhibited strong negative correlations with pro-inflammatory lipid mediators, specifically arachidonic acid and leukotriene B4. Meanwhile, some of them also showed significant negative correlations with purine metabolism-related markers, including hypoxanthine, xanthine, and inosine. These results suggest that these blood-absorbed components are potential active ingredients of CF, supporting its role in lowering uric acid and protecting the kidney by regulating arachidonic acid and purine metabolism pathways.

### 3.8. Molecular Docking of COX-1/PGE2/XOD & In Vivo/In Vitro Validation

**Figure 6:**
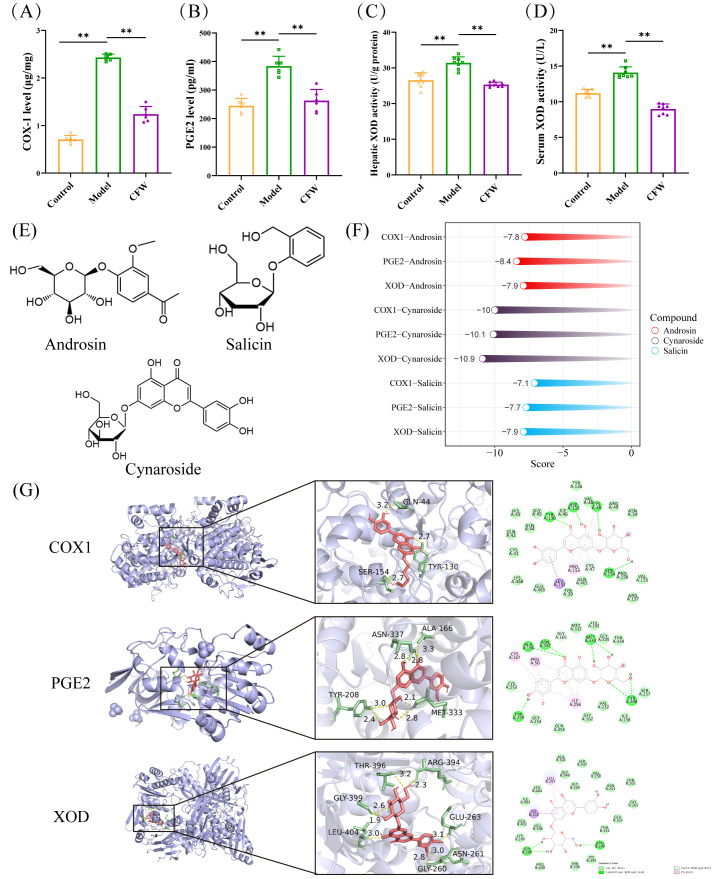
Experimental validation of therapeutic targets of CF and molecular docking analysis. (**A**,**B**) Results of enzyme immunoassay for COX-1 and PGE2 in kidney (n = 6). (**C**) The impact of CF on the hepatic XOD activity level. (**D**) Effect of CF on the level of XOD activity in serum (n = 8). (**E**) Structural formulae for androsin, cynaroside, and salicin. (**F**) Molecular docking scores of COX-1-1EQG, PGE2-2ZB4 and XOD-1FIQ with active compounds in CF (androsin, cynaroside and salicin). (**G**) The molecular docking results of cynaroside are shown Data are expressed as mean ± SD. ** *p* < 0.01 as compared with PO+HX model group.

We analyzed key indicators related to arachidonic acid metabolism and purine metabolism in the serum, kidneys, and liver of mice with hyperuricemia. The results demonstrated that CF significantly reduced the levels of COX-1 (1.23 ± 0.16 μg/mg) and PGE2 (262.85 ± 38.93 pg/mL) in the kidney (Figure 6A,B). Additionally, CF was found to markedly suppress the activity of XOD in both the serum and liver (9.22 ± 1.66 U/g and 25.18 ± 1.75 U/L) (Figure 6C,D).

Molecular docking analysis was conducted to explore the potential interactions between the primary blood-entry constituents of CF—androsin, cynaroside, and salicin and three key targets implicated in hyperuricemia: COX-1, PGE2, and XOD. The chemical structures of these compounds are shown in Figure 6E. Docking simulations suggested that all three compounds may exhibit favorable binding affinities toward the selected targets. Notably, cynaroside demonstrated the lowest binding energy values across multiple targets, indicating a potentially stronger predicted interaction (Figure 6F,G). Additionally, Indomethacin and Febuxostat were employed as positive controls targeting COX-1/PGE2 and XOD, respectively (Supplementary Figure S4).

**Figure 7:**
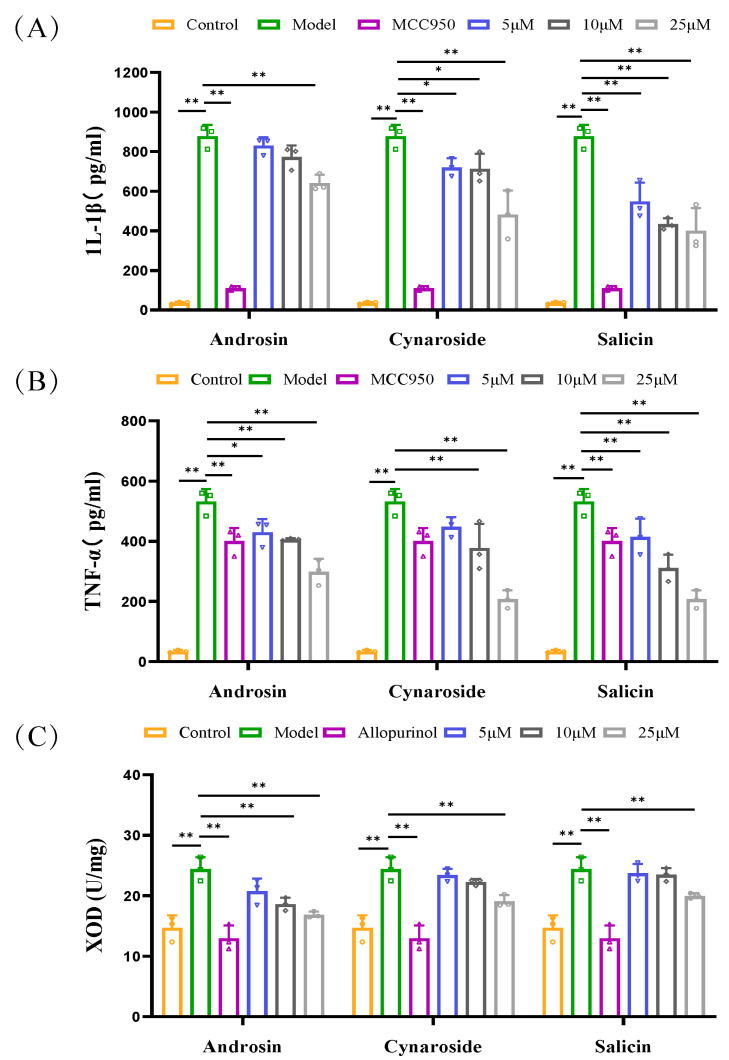
Regulatory effects of androsin, cynaroside, and salicin on inflammatory cytokines and XOD activity in in vitro models. (**A**,**B**) show the regulatory effects of androsin, cynaroside, and salicin on the levels of inflammatory cytokines IL-1β and TNF-α in the supernatant of bone marrow-derived macrophages (BMDMs) co-stimulated with LPS and MSU (n = 3). (**C**) The effects of androsin, cynaroside, and salicin on the levels of XOD protein activity in BRL3A cells under a xanthine environment (n = 3). * *p* < 0.05, ** *p* < 0.01 as compared with PO + HX model group.

To confirm the predicted interactions, the compounds were tested in relevant cell models. These three compounds did not exhibit toxicity to BMDM and BRL3A cells at concentrations ranging from 5 to 100 μM in vitro experiments (Supplementary Figure S5). At a concentration of 25 μM, androsin, cynaroside, and salicin significantly inhibited the elevation of IL-1β and TNF-α levels in BMDM cells stimulated by LPS + MSU, with cynaroside and salicin exhibiting the stronger anti-inflammatory effect. Additionally, these compounds effectively inhibited XOD activity in BRL3A cells under a xanthine environment at this concentration (Figure 7A–C). These findings highlight the multi-targeted mechanism of CF and its components in the treatment of hyperuricemia and underscore its potential as a therapeutic agent for this condition.

## 4. Discussion

Hyperuricemia is a complex metabolic disorder inextricably linked to chronic renal inflammation and oxidative stress [21]. While XOD inhibitors such as allopurinol remain the standard of care, their clinical utility is often compromised by adverse reactions, including hypersensitivity and potential renal toxicity, underscoring the urgent need for safer, multi-target natural alternatives [22,23,24]. In the present study, we employed an integrative strategy combining serum pharmacochemistry, metabolomics, and experimental validation to systematically decode the therapeutic mechanisms of CF. Our findings provide evidence that CF exerts anti-hyperuricemic and renoprotective effects through a coordinated multi-level mechanism: systemically restoring redox balance and regulating purine and arachidonic acid metabolism. Furthermore, androsin, cynaroside, and salicin were identified as key blood-absorbed bioactive components that likely serve as the material basis for these effects, potentially by inhibiting both XOD activity and the COX-1/PGE2 inflammatory axis.

Clinically, the primary therapeutic goal is to lower serum UA to preventing urate crystal deposition [25]. In our in vivo experiments, CF treatment elicited a potent, dose-dependent reduction in serum UA, CRE, and BUN levels, effectively restoring them toward baseline. These biochemical improvements were strongly corroborated by histopathological evidence, where CF treatment markedly reversed HUA-induced renal injuries, including glomerular atrophy, tubular dilation, and inflammatory cell infiltration. It is worth noting that the biochemical improvements in renal function were closely mirrored by the restoration of antioxidant capacity [26,27]. The observed upregulation of hepatic SOD and GSH-Px activities, coupled with the reduction in MDA content, suggests a potential mechanism by which CF may attenuate renal damage: namely, by interrupting the “oxidative stress–inflammation” vicious cycle, which in turn could help preserve the structural integrity of the kidney. Intriguingly, the positive control group (Allopurinol) exhibited paradoxically elevated CRE/BUN levels and aggravated renal pathology compared to the CF group, a phenomenon likely associated with the known nephrotoxicity or hypersensitivity risks of allopurinol [28,29], further highlighting the superior safety profile of CF at the effective dose.

To investigate the metabolic alterations associated with these phenotypic improvements, we employed untargeted metabolomics. The analysis indicated that CF treatment helps regulate perturbed metabolic networks, particularly involving purine catabolism and inflammatory lipid signaling. Notably, the metabolic adjustments observed in the CF group were consistent with the therapeutic outcomes. First, in the purine metabolism pathway, CF treatment was associated with a downregulation of serum hypoxanthine and xanthine levels. As these metabolites serve as direct substrates for uric acid generation, their reduction aligns with the observed decrease in serum UA and correlates with the inhibition of hepatic and serum XOD activity demonstrated in our enzymatic assays [30]. Second, regarding the arachidonic acid (AA) metabolism pathway [31], CF appeared to attenuate the accumulation of pro-inflammatory mediators, including arachidonic acid and leukotriene A4 (LTA4). Given the role of LTA4 as a chemotactic agent for immune cells, its downregulation is consistent with the histological observation of reduced inflammatory cell infiltration in renal tissues. Moreover, the modulation of metabolites such as phosphocholine and creatinine may reflect improvements in membrane phospholipid homeostasis and renal filtration function.

Transitioning from systemic metabolic regulation to the material basis, we focused on the constituents of CF absorbed into the blood, postulating them as the primary effectors in vivo. Through integrated bioinformatics and correlation analysis, androsin [32], cynaroside [33], and salicin [34,35] were identified as potential key bioactive candidates. Notably, our data proposes a novel “dual-inhibition” potential for these components. On one hand, molecular docking and in vitro enzymatic assays demonstrated that cynaroside and salicin bind deeply into the active pocket of XOD, directly inhibiting its activity; this interaction creates the molecular basis for the systemic reduction in purine metabolites (xanthine/hypoxanthine) and serum UA. On the other hand, these components were found to target the inflammatory axis by binding to COX-1 and PGE2. This was validated in BMDM cells, where the compounds significantly suppressed cytokine release (IL-1β, TNF-α), providing a cellular-level explanation for the alleviation of AA metabolism-driven renal inflammation.

Despite these promising findings, rigorous caution is warranted in interpreting the direct causality of specific components. While identifying androsin, cynaroside, and salicin as absorbed constituents provides a strong lead, their specific pharmacokinetic profiles—including bioavailability, half-life, and tissue distribution within the kidney—remain to be fully elucidated. Furthermore, although metabolomics highlighted the regulatory role of the AA pathway, the specific upstream molecular nodes (e.g., PLA2 activity or specific transporter expression) were not directly quantified in this study. Future investigations employing gene-knockout models or specific inhibitors are necessary to definitively validate the causal contribution of the COX-1/PGE2 pathway to CF-mediated renoprotection.

## 5. Conclusions

In summary, this study provides preliminary evidence that CF exerts uric acid-lowering and renoprotective effects in hyperuricemic mice, potentially through modulating purine metabolism, arachidonic acid metabolism, and oxidative stress pathways. The integrated approach of functional evaluation, metabolomics, and bioinformatics identified androsin, cynaroside, and salicin as potential bioactive components, which may target COX-1, PGE2, and XOD to inhibit inflammation and UA production. These results validate the traditional use of CF as a dietary component for managing hyperuricemia and provide a scientific basis for its development into functional foods. Nevertheless, more rigorous studies are needed to refine our understanding of CF’s mechanisms and optimize its clinical application.

## Figures and Tables

**Table 1:** Chemical composition of CF (Sort by relative peak area).

NO.	Formula	English Name	Class	Experimental *m*/*z*	Theoretical *m*/*z*	Mass Error (ppm)	Relative Peak Area	Retention Time (min)
1	C_15_H_14_O_6_	Catechin	Flavonoids	289.0724	289.0729	2.05	24.58134	6.91
2	C_16_H_18_O_9_	Chlorogenic acid	Organooxygen compounds	353.0886	353.0893	2.18	20.34949	7.23
3	C_16_H_18_O_9_	Neochlorogenic acid	Organooxygen compounds	353.0886	353.0893	2.15	17.95014	6.60
4	C_6_H_8_O_7_	Citric acid	Carboxylic acids and derivatives	191.0190	191.0182	−4.04	8.18370	0.91
5	C_15_H_20_O_8_	Androsin	Organooxygen compounds	327.1094	327.1102	2.50	4.59772	8.30
6	C_21_H_20_O_12_	Hyperoside	Flavonoids	463.0895	463.0909	2.88	3.75617	11.25
7	C_9_H_8_O_4_	Caffeic acid	Cinnamic acids and derivatives	163.0389	163.0389	−0.34	3.10316	7.30
8	C_27_H_30_O_16_	Rutin	Flavonoids	609.1481	609.1501	3.29	2.83269	10.93
9	C_7_H_7_NO_2_	Trigonelline	-	138.0550	138.0550	0.27	2.32087	0.86
10	C_6_H_6_O_3_	5-Hydroxymethylfurfural	Organooxygen compounds	127.0393	127.0397	2.74	1.62351	3.39
11	C_21_H_22_O_11_	Astilbin	Flavonoids	449.1101	449.1113	2.61	1.51919	11.27
12	C_7_H_12_O_6_	Quinic acid	Organooxygen compounds	237.0613	237.0609	−1.50	1.06812	0.86
13	C_21_H_22_O_12_	Plantagoside	Flavonoids	465.1046	465.1054	1.63	0.66332	10.36
14	C_17_H_24_O_11_	Deacetyl asperulosidic acid methyl ester	Prenol lipids	403.1255	403.1263	2.18	0.61402	5.70
15	C_7_H_10_O_5_	Shikimic acid	Organooxygen compounds	219.0506	219.0500	−2.59	0.61019	1.33
16	C_20_H_27_NO_11_	Amygdalin	-	502.1581	502.1598	3.29	0.57485	7.84
17	C_21_H_20_O_11_	Cynaroside	Flavonoids	447.0943	447.0954	2.32	0.51586	11.44
18	C_15_H_14_O_6_	Cianidanol	Flavonoids	335.0782	335.0793	3.35	0.49643	9.05
19	C_7_H_6_O_3_	Protocatechualdehyde	Organooxygen compounds	139.0391	139.0392	0.79	0.48969	5.95
20	C_30_H_48_O_3_	Oleanic acid	Prenol lipids	439.3569	439.3568	−0.23	0.42108	25.06
21	C_30_H_48_O_4_	Echinocystic acid	Prenol lipids	437.3415	437.3415	0.12	0.30144	22.58
22	C_6_H_12_O_6_	Inositol	Organooxygen compounds	225.0611	225.0605	−2.60	0.28229	0.86
23	C_10_H_18_O	Linalool	Prenol lipids	172.1695	172.1693	−0.82	0.24527	19.70
24	C_21_H_20_O_11_	Quercitrin	Flavonoids	447.0946	447.0958	2.83	0.22024	12.31
25	C_8_H_8_O_4_	Isovanillic Acid	Benzene and substituted derivatives	169.0495	169.0494	−0.26	0.21689	7.37
26	C_27_H_30_O_15_	Nicotiflorin	Flavonoids	593.1529	593.1546	2.83	0.19800	11.96
27	C_15_H_10_O_7_	Quercetin	Flavonoids	301.0360	301.0367	2.23	0.18502	15.38
28	C_5_H_5_N_5_O	Guanine	Imidazopyrimidines	152.0567	152.0567	0.18	0.17431	0.88
29	C_10_H_10_O_4_	Ferulic acid	Cinnamic acids and derivatives	177.0547	177.0547	0.18	0.16582	10.73
30	C_9_H_12_N_2_O_6_	Uridine	Pyrimidine nucleosides	243.0626	243.0630	1.44	0.15876	1.39
31	C_15_H_10_O_5_	Baicalein	Flavonoids	271.0598	271.0594	−1.26	0.14143	17.76
32	C_10_H_13_N_5_O_5_	Guanosine	Purine nucleosides	284.0989	284.0988	−0.19	0.13961	1.49
33	C_30_H_48_O_5_	Bayogenin	Prenol lipids	487.3441	487.3453	2.44	0.13117	20.71
34	C_20_H_22_O_8_	Piceid	Stilbenes	435.1307	435.1318	2.59	0.12641	10.69
35	C_6_H_14_N_4_O_2_	Arginine	Carboxylic acids and derivatives	175.1190	175.1191	0.33	0.10519	0.81
36	C_9_H_8_O_3_	p-Coumaric acid	Cinnamic acids and derivatives	147.0441	147.0441	0.16	0.09260	9.59
37	C_17_H_20_O_9_	4-O-Feruloylquinicacid	Organooxygen compounds	367.1043	367.1051	2.19	0.08311	9.62
38	C_30_H_46_O_4_	Glycyrrhetinic acid	Prenol lipids	471.3471	471.3474	0.50	0.07458	22.62
39	C_21_H_20_O_11_	Astragalin	Flavonoids	449.1080	449.1082	0.43	0.06845	12.31
40	C_30_H_48_O_3_	3-Epioleanolic acid	Prenol lipids	455.3540	455.3549	2.00	0.06272	25.04
41	C_13_H_18_O_7_	Salicin	Organooxygen compounds	331.1044	331.1054	3.19	0.05273	5.62
42	C_15_H_22_O	Germacrone	Prenol lipids	219.1743	219.1742	−0.41	0.04886	22.91
43	C_11_H_14_O_3_	Zingerone	Phenols	177.0910	177.0910	0.11	0.04547	12.72
44	C_7_H_6_O_4_	3,4-Dihydroxybenzoic acid	Benzene and substituted derivatives	137.0234	137.0235	0.50	0.03873	4.24
45	C_30_H_48_O_5_	Asiatic acid	Prenol lipids	487.3440	487.3450	2.20	0.03810	20.25
46	C_10_H_10_O_4_	Kakuol	Benzodioxoles	177.0546	177.0545	−0.25	0.03590	19.64
47	C_15_H_12_O_5_	Naringenin	Flavonoids	271.0618	271.0625	2.31	0.03266	16.65
48	C_11_H_12_O_4_	Ethyl caffeate	Cinnamic acids and derivatives	207.0656	207.0649	−3.36	0.03106	15.84
49	C_9_H_10_O_4_	3,5-Dimethoxy-4-hydroxybenzaldehyde	Phenols	183.0653	183.0655	0.84	0.02550	10.35
50	C_11_H_14_O_2_	Methyl eugenol	Benzene and substituted derivatives	179.1066	179.1066	−0.23	0.02341	19.85
51	C_10_H_12_O	Estragole	Phenol ethers	149.0961	149.0961	0.08	0.02174	20.52
52	C_21_H_20_O_10_	Sophoricoside	Isoflavonoids	433.1131	433.1133	0.40	0.02138	12.67
53	C_8_H_8_O_3_	Isovanilline	Phenols	153.0547	153.0547	0.23	0.01942	9.46
54	C_11_H_14_O_4_	Sinapyl alcohol	Phenols	193.0861	193.0862	0.64	0.01890	10.24
55	C_17_H_26_O_4_	6-Gingerol	Phenols	339.1818	339.1824	1.67	0.01849	19.58
56	C_11_H_12_O_4_	Methyl ferulate	Cinnamic acids and derivatives	209.0809	209.0809	0.18	0.01815	16.89
57	C_11_H_20_O	Undecenal	Organooxygen compounds	186.1852	186.1851	−0.44	0.01403	20.70
58	C_15_H_14_O_6_	L-Epicatechin	Flavonoids	273.0755	273.0753	−0.73	0.01228	8.28
59	C_15_H_24_O_2_	Dihydroartemisinic acid	Prenol lipids	219.1743	219.1743	−0.16	0.00895	22.38
60	C_13_H_18_O	(4E)-4-[(E)-But-2-enylidene] -3,5,5-trimethylcyclohex -2-en-1-one	Organooxygen compounds	191.1429	191.1428	−0.64	0.00859	20.61
61	C_11_H_12_O_5_	Sinapic Acid	Cinnamic acids and derivatives	207.0652	207.0653	0.17	0.00844	11.04
62	C_9_H_8_O_2_	Cinnamic acid	Cinnamic acids and derivatives	131.0494	131.0496	1.75	0.00690	15.43
63	C_8_H_18_O_5_	Tetraethylene glycol	Organooxygen compounds	159.1017	159.1017	0.44	0.00544	8.97

## Data Availability

The original contributions presented in this study are included in the article/Supplementary Materials. Further inquiries can be directed to the corresponding authors.

## Supplementary Materials

The following supporting information can be downloaded at: https://www.mdpi.com/article/10.3390/foods15010020/s1, Tables S1–S4. Parameter for chemical composition analysis of CF and serum. Tables S5–S8. Parameter for untargeted metabolomics analysis. Table S9. Differential metabolites in mouse serum metabolomics. Table S10. Criteria for renal histopathological scoring. Table S11. The grid box parameters. Figure S1. Base peak ions chromatogram of Incoming blood components detected in positive and negative mode. Figure S2. The chromatograms of serum metabolomics. A: representative chromatograms of CF samples in positive ion mode. B: Representative chromatograms of the negative ion patterns of CF samples. Figure S3. OPLS-DA analysis of non-targeted metabolomics of mouse serum and validation results. A: OPLS-DA score plots and validation diagrams (positive ions). B: OPLS-DA score plots and validation diagrams (negative ions). Figure S4. Molecular docking visualizations of positive control drugs with their respective targets. Figure S5. Cell viability of BMDM (A) and BRL3A (B) cells at different concentrations of androsin, cynaroside, and salicin.

## Abbreviations

CF	Chaenomeles Fructus	COX-1	cyclooxygenase-1
CF	Chaenomeles Fructus watery extract	XOD	xanthine oxidase
CRE	creatinine	PGE2	prostaglandin E2
UA	uric acid	PO	potassium oxonate
GSH-Px	glutathione peroxidase	HX	hypoxanthine
BUN	blood urea nitrogen	CMC-Na	sodium carboxymethyl cellulose
MDA	malondialdehyde	ALL	allopurinol
SOD	superoxide dismutase	CCK-8	Cell Counting Kit-8
LPS	lipopolysaccharide	MSU	Monosodium urate
BMDM	Bone marrow-derived macrophages	PBS	Phosphate-buffered saline
FBS	Fetal bovine serum	DMEM	Dulbecco’s modified eagle medium
BRL3A	Buffalo Rat Liver-3A		
