# Supplementary material for: Bioactive Components, Untargeted Metabolomics and Bioinformatics of Chaenomeles speciosa Fruit on Uric Acid-Lowering Activity Assessment
Source: Foods. 2025 Dec 22;15(1):20. doi: 10.3390/foods15010020 (PMC12785527; doi:10.3390/foods15010020)
Supplement: Supplementary file 1 [file foods-15-00020-s001.zip › foods-4017817-supplementary.pdf]

Supplementary data

1. Parameter for chemical composition analysis of CF and serum (Table. S1-S4).

Table S1.

Instruments

Name	Model	Manufacturer
Ultrasonic extractor	KQ3200D	Kunshan Ultrasonic Instruments Co., Ltd.
High-speed refrigerated centrifuge	Mikro 220R	Hettich Lab Technology
Reversed-phase column	ACQUITY UPLC HSS T3 (1.8 μm, 2.1 mm × 100 mm)	Waters Corporation
Ultra-high-performance liquid chromatograph	Vanquish Flex UHPLC	Thermo Fisher Scientific
Hybrid quadrupole orbitrap mass spectrometer	Q Exactive	Thermo Fisher Scientific

Table S2.

Reagents

Name	Grade	Article No.	Manufacturer
Water	LC-MS/4 L	W6-4	Thermo Fisher Scientific
Methanol	LC-MS/4 L	A456-4	Thermo Fisher Scientific
Acetonitrile	LC-MS/4 L	A955-4	Thermo Fisher Scientific
Formic acid	LC-MS/50 mL	A117-50	Thermo Fisher Scientific

Table S3.

Liquid chromatography elution gradient

Time (min)	Flow rate (mL·min–1)	Water with 0.1% formic acid (%)	Acetonitrile (%)	Sample volume (μl)	Column temperature (℃)
0	0.3	98	2	6	40
1.0	0.3	98	2	6	40
14.0	0.3	70	30	6	40
25.0	0.3	0	100	6	40
28.0	0.3	0	100	6	40
28.1	0.3	98	2	6	40
30.0	0.3	98	2	6	40

Table S4.

Mass spectrometry parameter

Metabolomics	Mice optimal conditions	
	Positive mode	Negative mode
Ion source voltage (kV)	3.7	3.5
Capillary heating temperature (°C)	320	320
Sheath gas pressure (pis)	30	30
Auxiliary gas pressure	10	10
Solvent heating and evaporation temperature (°C)	300	300
Sheath gas and auxiliary gas	Nitrogen gas	Nitrogen gas
Collision gas	Nitrogen gas	Nitrogen gas
Collision gas pressure (mTorr)	1.5	1.5
Acquisition mode	Xcalibur	Xcalibur

2. Parameter for untargeted metabolomics analysis (Tables. S5-S8).

Table. S5

Instruments

Name	Model	Manufacturer
Ultra-Fast Liquid Chromatograph	Dionex UltiMate 3000	Thermo Scientific
High-speed refrigerated centrifuge	Mikro 220R	Hettich Lab Technology
Reversed-phase column	ACQUITY UPLC HSS T3 (1.8 μm, 2.1 mm × 100 mm)	Waters Corporation
Vacuum centrifuge concentrator	Savant SPD131DDA SpeedVac	Thermo Scientific
Hybrid quadrupole orbitrap mass spectrometer	Q Exactive	Thermo Fisher Scientific

Table. S6

Reagents

Name	Grade	Article No.	Manufacturer
Water	LC-MS/4 L	W6-4	Thermo Fisher Scientific
Methanol	LC-MS/4 L	A456-4	Thermo Fisher Scientific
Formic acid	LC-MS/50 mL	A117-50	Thermo Fisher Scientific

Table. S7

The optimal UPLC linear gradients.

Time (min)	Flow rate (mL·min–1)	A: Water with 0.1% formic acid (v%)	B: methyl alcohol (v%)	Sample volume（μl）	Column temperature (℃)
0	0.3	98	2	4	50
1.0	0.3	98	2	4	50
5.5	0.3	0	100	4	50
14	0.3	0	100	4	50
14.1	0.3	98	2	4	50
16.0	0.3	98	2	4	50

Table. S8

Mouse blood entry components.

No.	Name of Prototype	Transformations	Compound Name	Ion Mode	Adducts	Theoretical *m/z*	Mass Error (ppm)	Retention time (min)	Experimental *m/z*
1	4-O-Feruloylquinic acid		4-O-Feruloylquinic acid	NEG	M-H	367.1035	1.43	9.65	367.1040
2	4-O-Feruloylquinic acid	+C6H8O6	4-O-Feruloylquinic acid M1	NEG	M-H	543.1355	2.18	7.31	543.1367
3	Androsin		Androsin	NEG	M-H	327.1085	2.03	8.33	327.1092
4	Androsin	+CO	Androsin M1	NEG	M+FA-H	401.1089	1.40	6.23	401.1095
5	Androsin	-H4	Androsin M2	NEG	M+FA-H	369.0827	1.50	11.21	369.0833
6	Androsin	+O	Androsin M3	NEG	M-H	343.1035	1.32	6.24	343.1039
7	Androsin	+O2	Androsin M4	NEG	M-H	359.0984	2.31	5.70	359.0992
8	Astilbin	+SO3	Astilbin M1	NEG	M+FA-H	575.0712	2.16	6.02	575.0725
9	Astilbin	+C6H8O6	Astilbin M2	NEG	M-H	625.1410	2.55	7.92	625.1426
10	Chlorogenic acid		Chlorogenic acid	NEG	M-H	353.0878	1.96	7.56	353.0885
11	Cianidanol	+H2O	Cianidanol M1	NEG	M+FA-H	353.0878	1.78	5.54	353.0884
12	Cianidanol	+SO3	Cianidanol M2	NEG	M-H	369.0286	1.40	6.53	369.0291
13	Cynaroside	+H2	Cynaroside M1	NEG	M-H	449.1089	2.05	8.65	449.1099
14	Cynaroside	+O	Cynaroside M2	NEG	M-H	463.0882	2.59	8.47	463.0894
15	Deacetyl asperulosidic acid methyl ester		Deacetyl asperulosidic acid methyl ester	NEG	M-H	403.1246	1.63	5.74	403.1252
16	Deacetyl asperulosidic acid methyl ester	-H2O	Deacetyl asperulosidic acid methyl ester M1	NEG	M-H	385.1140	1.95	8.88	385.1148
17	Ethyl caffeate	+C6H8O6	Ethyl caffeate M1	NEG	M-H	383.0984	2.21	12.18	383.0992
18	Plantagoside	+H2O	Plantagoside M1	NEG	M+FA-H	529.1199	1.94	6.81	529.1209
19	Plantagoside	+SO3	Plantagoside M2	NEG	M-H	545.0607	2.21	6.44	545.0619
20	Quercitrin		Quercitrin	NEG	M-H	447.0933	2.23	12.58	447.0943
21	Salicin		Salicin	NEG	M+FA-H	331.1035	2.25	5.66	331.1042
22	Salicin	+O-H2	Salicin M1	NEG	M-H	299.0772	-0.20	2.78	299.0772
23	Salicin	-H2+O2	Salicin M2	NEG	M-H	315.0722	2.54	2.72	315.0730
24	Trigonelline		Trigonelline	POS	M+Na	160.0369	1.99	0.93	160.0372

Table S9.

Differential metabolites in mouse serum metabolomics.

Title	Metabolites	Pvalue	Log2Foldchange	VIP	FDR
1	2-Hydroxymyristoylcarnitine	0.00016	0.906204781	1.77538	0.01768
2	3-Hydroxy-11Z-octadecenoylcarnitine	0.00031	1.297426627	1.57954	0.03344
3	Leukotriene A4	0.00047	-0.903685598	1.57126	0.01952
4	Hypoxanthine	0.00062	-2.411760074	1.55014	0.02440
5	Arachidonic acid	0.00062	-2.03594463	1.53937	0.00995
6	3-Hydroxyhexadecanoylcarnitine	0.0011	0.898024228	1.53797	0.01880
7	Inosine	0.022	-2.57435933	1.51203	0.10950
8	Xanthine	0.033	-2.325676062	1.47443	0.02392
9	Tetradecanoylcarnitine	0.0047	-0.183880621	1.40904	0.03223
10	3, 5-Tetradecadiencarnitine	0.0065	-0.862730914	1.40124	0.03900
11	alpha-Linolenic acid	0.007	-3.845572375	1.3937	0.03733
12	PE(18:1/0:0)	0.01	-1.583328355	1.35673	0.02480
13	Deoxyadenosine	0.01	-0.403982114	1.34809	0.04364
14	Pantothenic acid	0.038	-1.26249615	1.33642	0.07600
15	Argininosuccinic acid	0.038	-1.672361651	1.31868	0.07296
16	Leukotriene B4	0.038	-1.391388883	1.29201	0.08291
17	Creatinine	0.031	-2.037835578	1.28652	0.08267
18	Phosphorylcholine	0.015	-0.664347324	1.27783	0.06000
19	N-oleoyl phenylalanine	0.021	-1.314144999	1.26943	0.07754
20	N-Arachidonoylglycine	0.021	-1.023367358	1.24491	0.07200
21	N-palmitoyl phenylalanine	0.039	-0.753286224	1.23876	0.06933
22	N-docosahexaenoyl phenylalanine	0.028	-0.62443657	1.23172	0.07906
23	N-stearoyl GABA	0.026	-0.482827662	1.22691	0.07800
24	Oleoyl glycine	0.042	-1.106724747	1.22305	0.04720
25	trans-2-Tetradecenoylcarnitine	0.032	-1.076988581	1.22143	0.08084
26	Oleoylethanolamide	0.024	-1.503963818	1.22065	0.04680
27	PE(16:1/0:0)	0.042	-1.282634785	1.19398	0.05300
28	PE(20:5/0:0)	0.043	-1.568278536	1.19202	0.06503
29	Oleoylcarnitine	0.037	-1.485855516	1.17278	0.08457
30	N-palmitoyl glycine	0.05	-1.1943557	1.16596	0.15217
31	Dodecanoylcarnitine	0.042	-1.167974763	1.15974	0.05600
32	Decanoylcarnitine	0.037	-0.878161936	1.15959	0.05760
33	Myristoleic acid	0.05	-2.562818221	1.15215	0.19212
34	L-Tryptophan	0.049	-2.274311213	1.13361	0.06034
35	9-Hexadecenoylcarnitine	0.048	-2.109076575	1.11661	0.07029
36	Stearidonic acid	0.044	-3.067491151	1.10288	0.05867
37	LysoPC(18:3/0:0)	0.044	-2.064985766	1.09911	0.06333
38	L-Palmitoylcarnitine	0.041	-1.917473815	1.09871	0.09470
39	Prostaglandin F2a	0.044	-2.087412605	1.09762	0.05811
40	LysoPC(22:5/0:0)	0.05	-1.979835944	1.08466	0.08785
41	Myristic acid	0.049	-3.388245533	1.08312	0.07200

42	7-Methylguanine	0.046	-3.293958865	1.07783	0.05838
43	Palmitolinoleic acid	0.047	-4.050638502	1.06941	0.10455
44	LysoPC(20:5/0:0)	0.039	-3.355602986	1.06938	0.06255
45	Palmitelaidic acid	0.045	-4.487539638	1.05568	0.12952
46	Butenylcarnitine	0.05	-7.768050833	1.03188	0.16195
47	Eicosapentaenoic acid	0.043	-3.804140454	1.02946	0.07930
48	Proline betaine	0.042	-3.096377565	1.02313	0.07106

Table S10.

Criteria for renal histopathological scoring

Parameter	Score	Description
Tubular dilation / distension	0	No dilation
	1	<25 % of tubules dilated
	2	25–50 % of tubules dilated
	3	50–75 % of tubules dilated
	4	>75 % of tubules dilated
Loss of brush border	0	Intact brush border in all proximal tubules
	1	<25 % of proximal tubules affected
	2	25–50 % affected
	3	50–75 % affected
	4	>75 % affected or complete loss
Tubular cast formation	0	No casts
	1	Casts in <25 % of tubular profiles
	2	25–50 %
	3	50–75 %
	4	>75 % or nearly all tubules contain casts
Interstitial inflammation	0	No inflammatory cells
	1	Few scattered inflammatory cells
	2	Mild focal infiltrates
	3	Moderate infiltrates with some disruption of architecture
	4	Severe, diffuse inflammation with loss of normal architecture
Interstitial fibrosis	0	No fibrosis
	1	<10 % fibrotic area
	2	10–25 %
	3	25–50 %
	4	>50 % fibrotic area
Total injury score		Sum of the five parameters (maximum 20)

Table S11.

The grid box parameters

Docking	Score	center_x	center_y	center_z	size_x	size_y	size_z
COX1-Androsin	-7.8	47.037	33.486	187.543	115.850	110.333	93.783
COX1-Cynaroside	-10	47.037	33.486	187.543	115.850	110.333	93.783
COX1-Salicin	-7.1	52.328	37.069	184.520	99.667	76.411	81.394
COX1-Indomethacin	-8.0	47.037	36.367	187.347	103.433	74.900	83.817
PGE2-Androsin	-8.4	15.932	33.096	56.261	54.167	53.083	68.250
PGE2-Cynaroside	-10.1	15.932	33.096	56.261	54.167	53.083	68.250
PGE2-Salicin	-7.7	15.932	33.096	56.261	54.722	53.628	68.950
PGE2-Indomethacin	-9.2	17.618	36.774	58.217	44.417	49.833	68.250
XOD-Androsin	-7.9	27.459	37.573	101.434	106.050	106.050	95.950
XOD-Cynaroside	-10.9	26.829	33.081	101.434	96.950	96.950	95.411
XOD-Salicin	-7.9	26.829	33.081	101.434	96.950	96.950	95.411
XOD-Febuxostat	-8.5	28.690	34.157	101.434	94.150	94.150	76.217

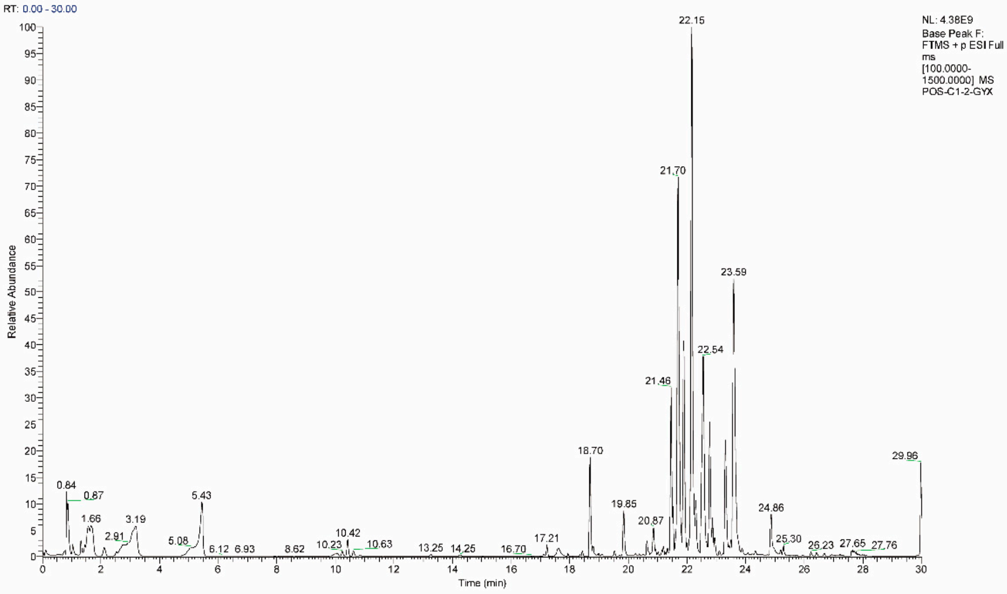

ESI(+)

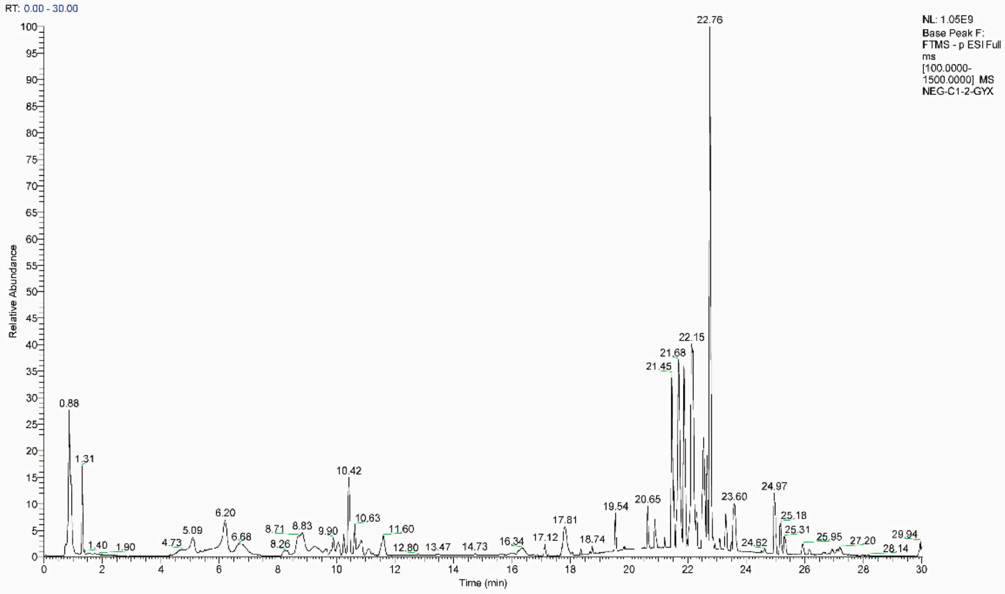

ESI(-)

Figure S1. Base peak ions chromatogram of Incoming blood components detected in positive and negative mode.

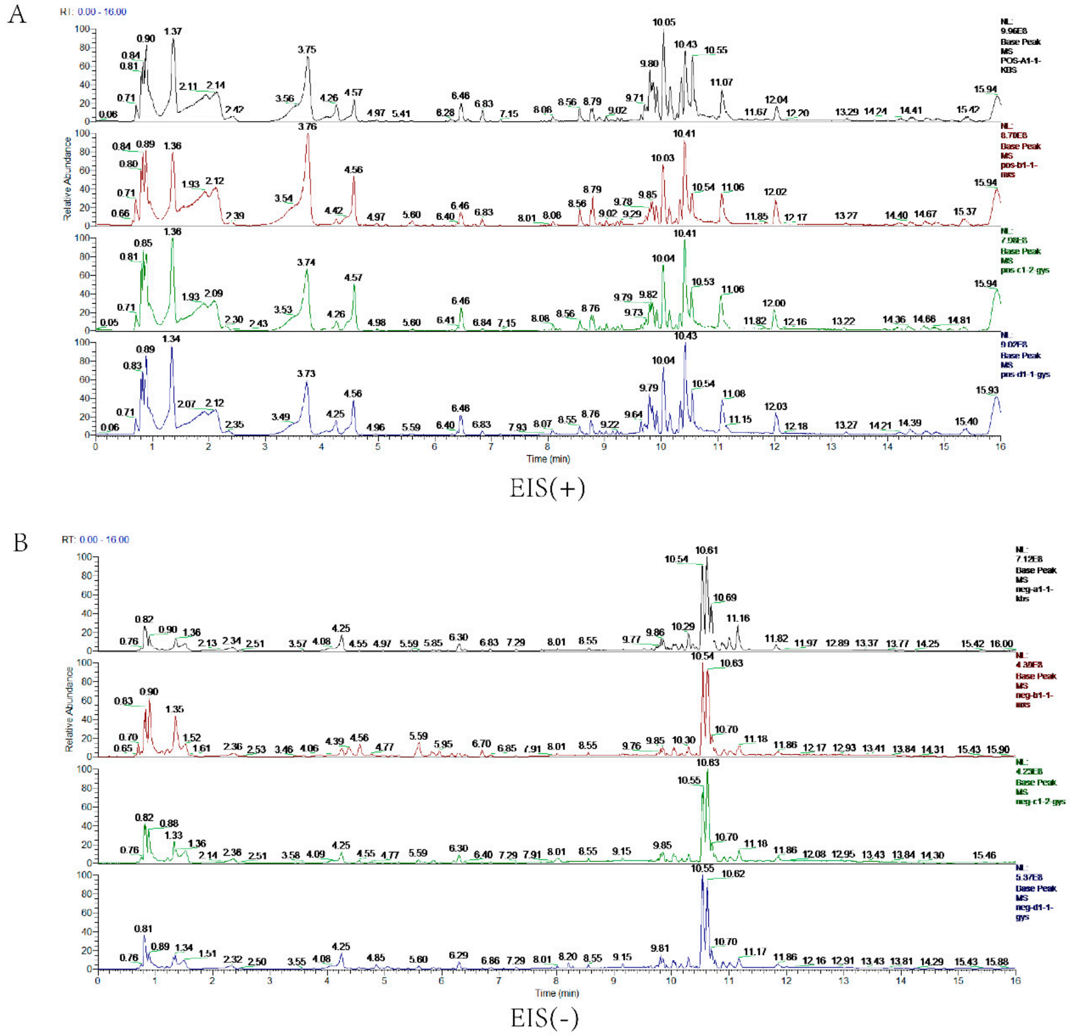

Figure S2. The chromatograms of serum metabolomics. A: representative chromatograms of CF samples in positive ion mode. B: Representative chromatograms of the negative ion patterns of CF samples.

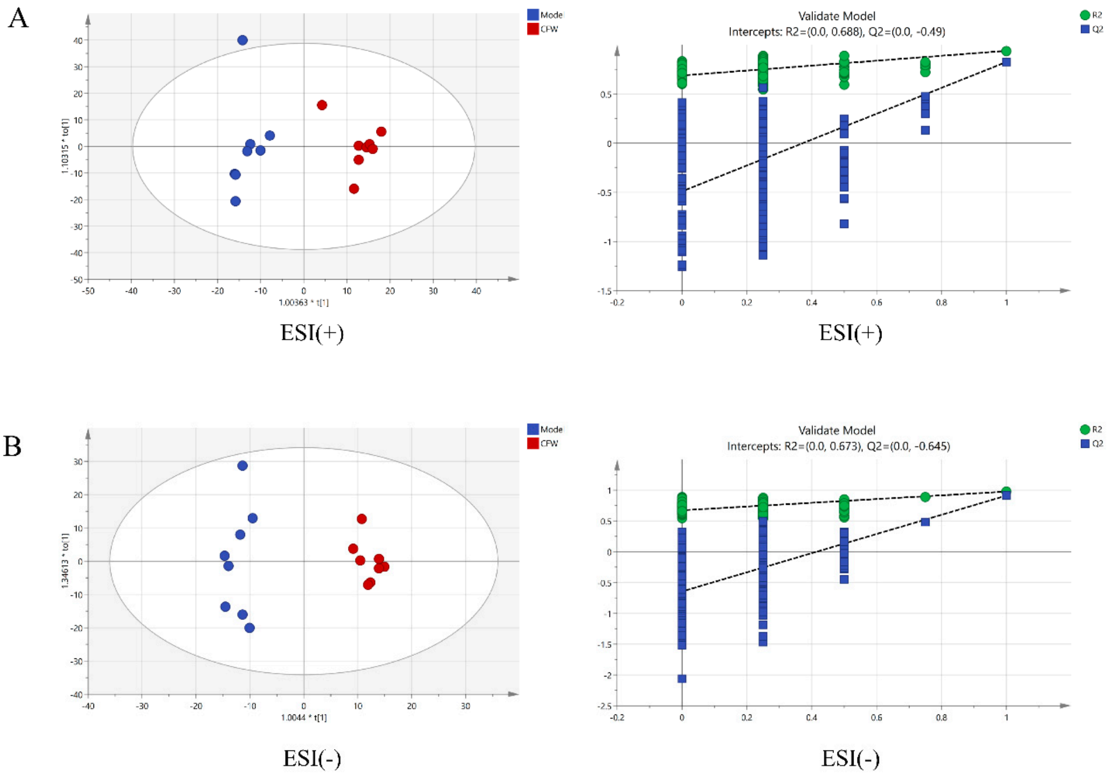

Figure S3. OPLS-DA analysis of non-targeted metabolomics of mouse serum and validation results. A: OPLS-DA score plots and validation diagrams (positive ions). B: OPLS-DA score plots and validation diagrams (negative ions).

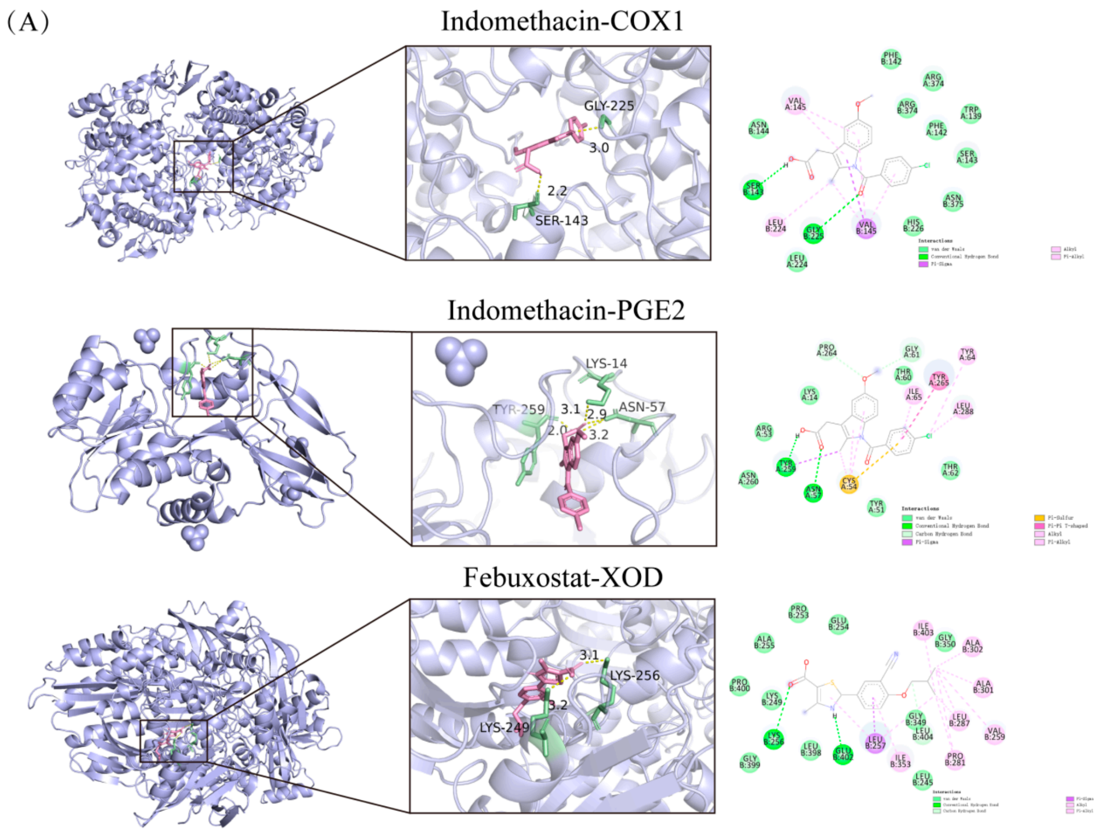

Figure S4. Molecular docking visualizations of positive control drugs with their respective targets. The binding modes of Indomethacin with COX-1 (top row) and PGE2 (middle row), and Febuxostat with XOD (bottom row) are displayed. The panels illustrate the overall 3D complex (left), the detailed binding pockets showing amino acid residues and hydrogen bond distances (middle), and the 2D interaction diagrams (right). These results serve as benchmarks for evaluating the binding performance of the test compounds.

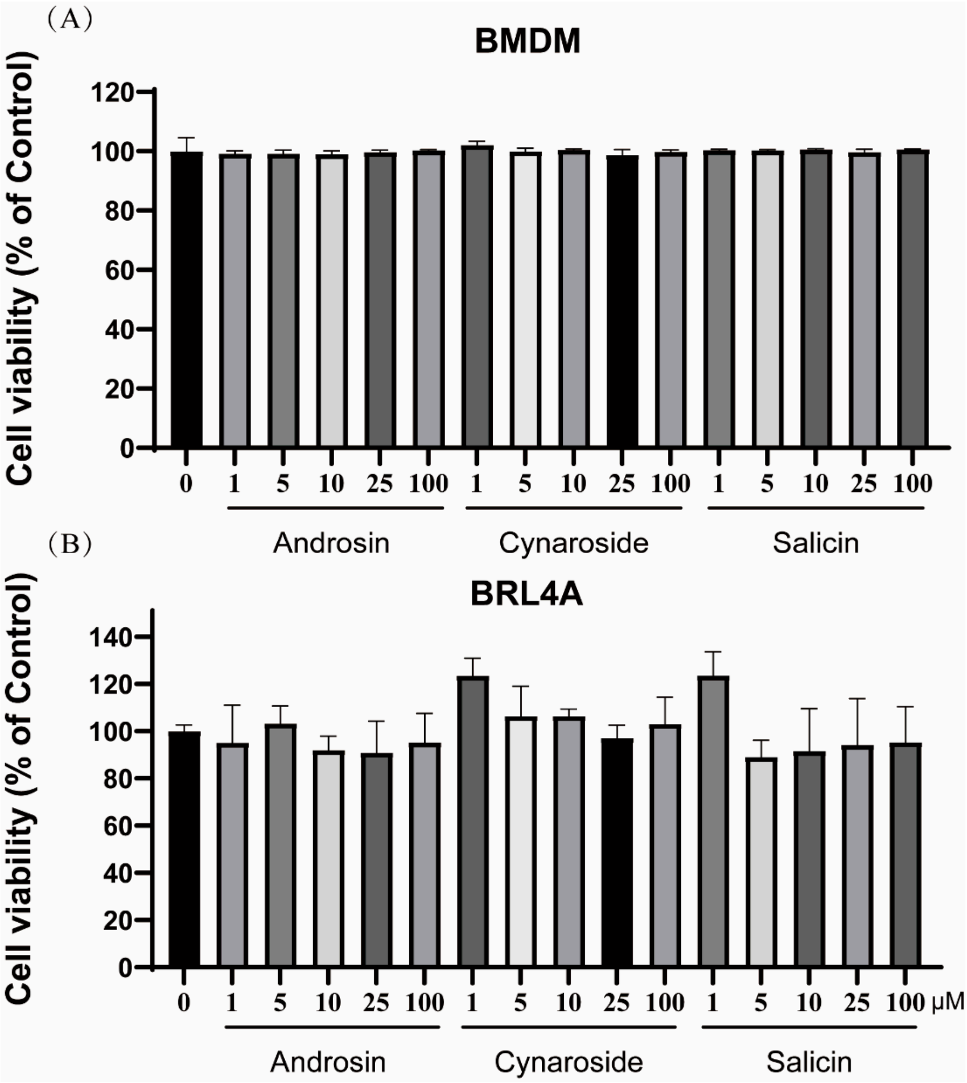

Figure S5. Cell viability of BMDM (A) and BRL3A (B) cells at different concentrations of androsin, cynaroside, and salicin.
